# Prevalence of Peri-Implant Disease in Patients with Stage III or IV Periodontitis: Results of a Long-Term Follow-Up from a Specialized Periodontal Practice

**DOI:** 10.3390/jcm12175547

**Published:** 2023-08-25

**Authors:** Pia-Merete Jervøe-Storm, Michael Marder, Martin Hagner, Ina Menn, Philipp Menn, Søren Jepsen

**Affiliations:** 1 Department of Periodontology, Operative and Preventive Dentistry, University Hospital Bonn, Welschnonnenstrasse 17, 53111 Bonn, Germany; soeren.jepsen@ukbonn.de; 2 Department of Prosthodontics, Preclinical Education and Dental Material Science, University Hospital Bonn, Welschnonnenstrasse 17, 53111 Bonn, Germany; michael.marder@ukbonn.de; 3 Practice for Interdisciplinary Dentistry, Im Mühlenbach 2b, 53127 Bonn, Germany; martin.hagner@gmx.net; 4 Practice for Interdisciplinary Dentistry Dr. Menn, Dammstraße 4, 57271 Hilchenbach, Germany; ina.dettmer@gmx.de (I.M.); philipp.menn@gmx.de (P.M.)

**Keywords:** dental implant, periodontitis, peri-implant health, peri-implant disease, periodontal supportive therapy

## Abstract

The aim of this study was to examine the conditions of implants that had been in function for 5–17 years in stage III/IV periodontitis patients of a specialized periodontal practice. There were 83 patients (43 female/40 male, mean age 64.4 (9.69) years), with a total of 213 implants, who participated in the study. Assessments included periodontal and peri-implant probing depths, bleeding and plaque scores, and a radiographic examination. Smoking habits, participation in a supportive care program (SCP), and the Implant Disease Risk Assessment (IDRA) scores were recorded. A total of 44 patients presented with stage III periodontitis, and 39 with stage IV. In all, 85% of patients had adhered to regular SCP. On an implant/patient level, peri-implant health was found in 37.1.7% (79 implants)/24.1% (20 patients), peri-implant mucositis in 58.7% (125 implants)/66.3% (55 patients), and peri-implantitis in 4.2% (9 implants)/9.6% (8 patients). IDRA scores showed 30.5% of implants at moderate and 69.5% at high risk. The present long-term analysis shows a high prevalence of peri-implant disease in patients treated for advanced periodontitis. These findings underline the challenges involved in the long-term maintenance of oral health in stage III/IV periodontitis patients restored with dental implants.

## 1. Introduction

Since the initial description of osseointegration in 1969 [1], a wealth of studies concerning both advantages and complications linked to treatment with dental implants have been published [2]. The replacement of teeth with dental implants is currently recognized as a standard treatment, allowing patients with tooth loss to achieve the restoration of function, aesthetics, and phonetics [2]. Also, from a patients’ point of view, treatment with oral implants is an interesting alternative to total or partial edentulism [3]. In contrast to wearing a complete denture, implant-supported restorations—irrespective of fixed or removable type, present a significant difference for most patients, particularly concerning quality of life [4]. A prospective study over 10 years found that more than 90% of 104 patients were completely satisfied with implant therapy, both from a functional and aesthetic point of view [5].

However, placing implants in patients with a history of periodontitis can be challenging, as peri-implant tissues are susceptible to the same dynamics that are believed to cause periodontitis [6,7]. A 10-year cohort study from a specialist periodontal practice demonstrated that periodontally healthy patients showed a significantly lower number of implants requiring additional treatment in comparison to implants in patients with a history of periodontitis [8]. Very recently, the 20-year follow-up data was presented [9]. The implant survival rate was 93%, and the odds of implant loss were almost eight times higher in non-compliant patients than in compliant patients [9]. One factor which may be considered in the planning of treating a patient with implants is the peri-implant microbiota, which shows a different ecosystem than the periodontal microbiota. A narrative review described the peri-implant microbiota to be less variable than the periodontal, but due to changes from peri-implant health to peri-implantitis, it becomes increasingly complex [10].

The criteria for success is not the same as the survival rate of the implants. The survival rate only describes whether the implant remains in the mouth at the examination time [11]. In 2018, results from World Workshop on the Classification of Periodontal and Peri-Implant Diseases and Conditions were published. For the first time, experts from all over the world reached a consensus, not only regarding various periodontal conditions, but also concerning clinical case definitions for peri-implant health, peri-implant mucositis, and peri-implantitis [12,13]. Peri-implant health is characterized by the absence of erythema, bleeding on probing, swelling, and suppuration. A range of probing depths linked to health, as in the case of natural teeth, cannot be defined for implants [12].

Treatment of periodontal, as well as peri-implant, diseases ranges from nonsurgical to surgical methods, with or without the use of adjunctive devices or agents. Peri-implant mucositis, as well as gingivitis, are treated successfully with nonsurgical methods, and the choice of therapy for periodontitis or peri-implantitis depends on the severity of the stage of disease. Additional adjunctive therapies can be considered, and in each case, the decision is made according to each patient’s needs and history [14,15,16,17].

Most published results regarding implant therapy in periodontally compromised patients originate from studies conducted in university settings under ideal conditions. At the Sixth European Workshop on Periodontology 2008, the necessity to recruit patients from private or public dental clinics, and not from university hospitals, was expressed and requested [18].

Recently, an Implant Disease Risk Assessment tool (IDRA) was presented [19]. Similar to the periodontal risk assessment (PRA) developed for patients in supportive periodontal therapy (SPT) [20], a tool for assessing the risk for an individual patient of developing of peri-implant disease after implant therapy was proposed. Through evaluation of eight risk factors associated with implant therapy, the risk for development of peri-implant disease can be calculated [19].

The aim of the present long-term follow-up study, which was carried out in a specialized periodontal practice, was to evaluate the prevalence of peri-implant health and disease in implants which had been in function for at least 5 years. The prevalence of peri-implant mucositis and peri-implantitis, as well as possible patient and implant factors which could influence peri-implant health or peri-implant disease, were additionally investigated. Moreover, the IDRA tool was used to evaluate the risk for further peri-implant disease for each implant.

## 2. Materials and Methods

### 2.1. Study Design

**Figure 1:**
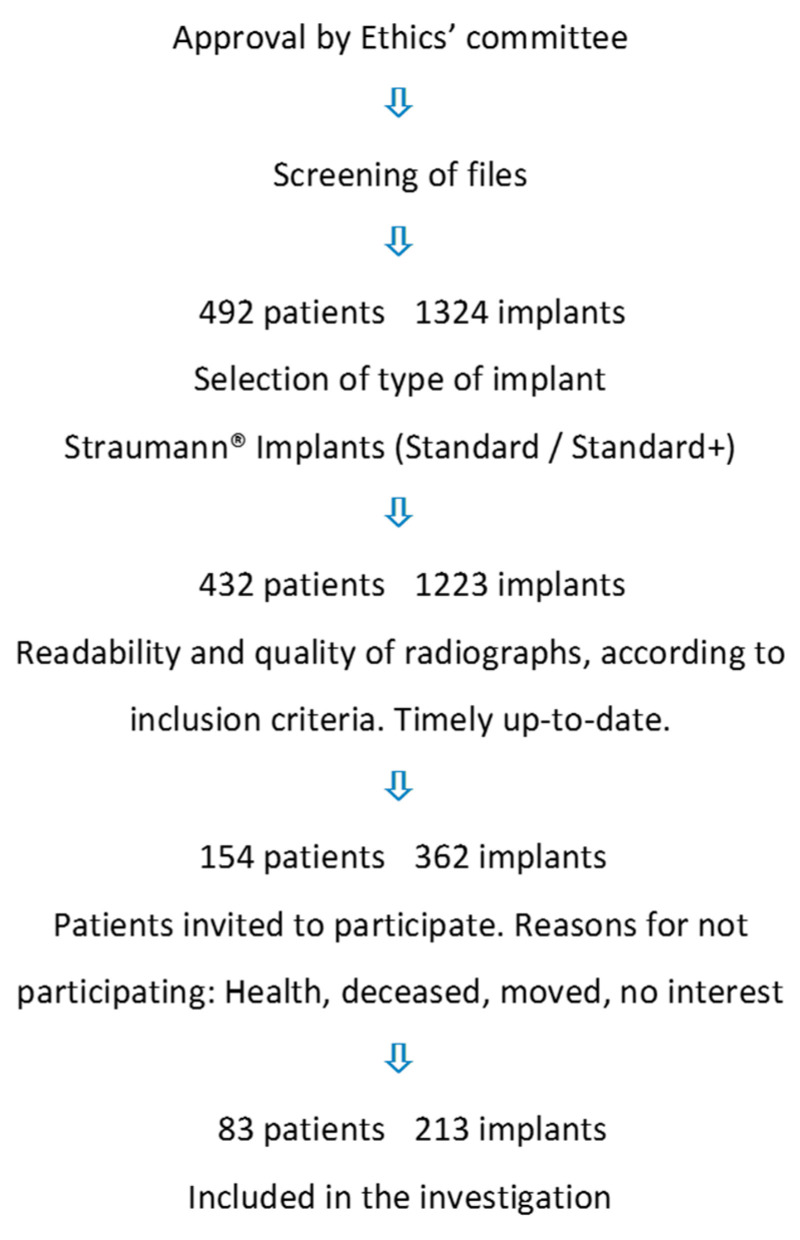
Flowchart of screening of patients.

The present investigation was designed as an observational study, carried out in a private dental practice specializing in periodontology and implantology. The flow chart shows the selection of patients for the study (Figure 1). After inclusion in the study, patients underwent a clinical examination of their teeth and implants, and radiographs were only obtained if indicated from a clinical point of view. All subjects provided informed consent for inclusion before they participated in the study. The university’s ethics committee approved the study (University of Bonn, Germany; ID: 157/10; date of approval 29 July 2010). The study was conducted in accordance with the European directives and ICH Harmonized Tripartite Guideline E6: Note for Guidance on Good Clinical Practice, CPMP/ICH/135/95 Step 5 (http://www.ema.europa.eu/ema/, accessed 2 April 2019), as well as in compliance with the Declaration of Helsinki (2013 Brazil).

### 2.2. Population

A screening of possible candidates for the study was based on patient files. A screening of files from patients treated with implants resulted in 492 patients with 1324 implants. After inclusion and exclusion criteria were clarified, patients were invited, in writing, to participate in the study. The patients received detailed information about possible risks and benefits of the clinical examination, signed an informed consent before enrollment in the study, and provided their approval to receive a clinical examination and to have their radiographs screened. (Figure 1).

Inclusion criteria:

- Treatment with a Straumann^®^ tissue-level implant (Standard/Standard Plus; Straumann^®^, Basel, Switzerland), with the surface of the implants coated with either SLA^®^- or SLActive^®^.
- One radiograph from the time point of insertion and one from an arbitrary later date should be available. The last radiographs should be up-to-date, from within one year prior to examination.

Exclusion criteria:

- Insufficient radiographs;
- Other types of implants from the same manufacturer, or other brands of implants.
- Missing or unanalyzable radiographs, or radiographs failing to display the implant in full length.

### 2.3. Clinical Examination

After signing the consent, the medical history was obtained and a questionnaire as to smoking habits was completed. Patients were sub-grouped into “smokers” and “former smokers/non-smokers”. Former smokers were defined as patients who had stopped smoking at the time of insertion of the implant, resulting in no smoking for at least the last 5 years [21]. For each patient, a full-mouth-plaque-index (FMPS [22]), full mouth bleeding score (FMBS), probing pocket depths (PPD), clinical attachment level (CAL), and gingival recessions (GR) were recorded using a manual periodontal probe (PCP12, HuFriedy, Frankfurt am Main, Germany) at six sites per tooth (mesio-vestibular, vestibular, disto-vestibular, mesio-oral, oral, and disto-oral); the cementoenamel junction was used as reference for measurements of CAL. The implants were assessed in the same fashion: at six sites, plaque, bleeding on probing (BOP), and purulence (all dichotomously) were concomitantly recorded with probing depths (PD), along with the distance between the implant shoulder and the mucosal margin (DIM) [23]. The latter measurement is synonymous with the measurement of a recession at the tooth. A subgingival implant shoulder yields a negative value of DIM.

The site of the implant displaying the greatest bone loss, based on radiographs, was defined as study site for the implant. The width of the keratinized mucosa (masticatory mucosa) at the implants was measured, buccal and lingual in the lower jaw and buccal in the maxilla; for an analysis, the two groups were composed of a <2 mm or ≥2 mm zone of keratinized mucosa [24]. Additionally, the compliance of the patients was determined based on the regularity of their maintenance visits. Patients were sub-grouped into “patients coming in regularly, at least once a year” and “patients who had no regular maintenance at all, or came in sporadically, at best”. Periodontal health, as well as the severity of the periodontal disease, was determined based on clinical measurements and radiological findings [25]. The diagnosis of peri-implant health [26], peri-implant mucositis [27], and peri-implantitis [28] was based on clinical and radiological findings. One examiner measured all clinical parameters, and another examiner evaluated the radiographs. Intra-examiner agreement level within 1 mm (±1 mm) was 95%.

For the evaluation of the Implant Disease Risk Assessment (IDRA), the history of periodontitis (yes/no); BOP (%); PPD ≥ 5 mm; bone level/age (BL/Age); “perio susceptibility”, using staging and grading [25]; frequency of SPT; RM–bone (distance restorative margin to marginal bone crest); and the factors of the restoration (cleanable, supra- or subgingival poor fit, excess cement, not cleanable) were assessed. By using the link to the online tool obtained from the work of Heitz-Mayfield et al. [19] (http://www.ircohe.net/IDRA, accessed on 18 July 2023), the calculation was performed for each implant. If different implants yielded different scores within one patient, the highest score was chosen as the score for that patient [29].

Peri-implant health was adapted from the definitions of the 2017 World Workshop on the Classification of Periodontal and Peri-Implant Diseases and Conditions [12]. On a patient level, success of treatment was acknowledged if the adjacent tissues around of the patient’s implants were considered as healthy. Peri-implant conditions around an implant was designated to be clinically healthy if the following criteria were fulfilled: absence of bleeding and/or suppuration upon gentle probing; no increase in probing depth compared to that found at previous examinations; and the absence of bone loss beyond the crestal bone level changes resulting from the initial bone remodeling [12].

### 2.4. Radiographic Examination

Radiographic examinations were performed to obtain a standard immediately after implant surgery. Standardized digital intraoral radiographs as panoramic images were also used for the evaluation. The radiographs were obtained using a Progeny Dental (Midmark Corporation, Versailles, OH, USA) device, exported from the practice software CharlyXL^®^ (Solutio GmbH, Holzgerlingen, Germany), and evaluated and measured using ImageJ (Institute of Health). For initial calibration of the radiographs, the implant thread was measured using the “Analyze/Set Scale” function of the ImageJ measurement program. The implant thread for the Straumann^®^ tissue-level implant Standard and Standard Plus is 1 mm at implants with a diameter of 3.3 mm. All other implants of those two types have an implant thread of 1.25 mm. Secondly, the distance between the most crestal point of the first bone to implant contact (BIC) at each of the 213 implants, as well as the tip of the implant, was measured at the mesial and distal aspects. With the function “Analyze/Measure”, the markings could be determined with a precision of 0.01 mm. Third, the mean of the mesial and distal measurements per implant was calculated for each radiograph, and the differences between the radiograph from the time of insertion and the latest radiograph was defined as bone loss.

### 2.5. Statistical Analysis

Mainly descriptive statistics were obtained, including descriptive summary statistics with the mean and a 95% confidence interval (95% CI), standard deviation (SD), and median. Associations between implant specific outcome “% of BOP at implants” (% positive sites at implants) and teeth-related measurements, including “% of BOP at teeth” (% positive sites at teeth) or “% of residual pockets at teeth ≥5 mm”, were analyzed with Kendall’s Tau-b, in which a causal relationship could not be readily assumed. For detection of potentially causal relationships, a binary logistic regression model was fit at the patient level, with the “peri-implant condition” (healthy/inflammation) as the outcome variable. The outcome for patients with multiple implants was aggregated, and the highest score for “peri-implant condition” represented the patient diagnosis, as is usually the case for the classification of peri-implant diseases [29]. “Gender”, “smoking status”, “SPT compliance”, “number of lost teeth”, “patient age at examination”, “FMPS” and “total number of implants per patient” were included in the model as predictors.

Additionally, to account for a potential correlation among measurements at implants from within the same patient, a multilevel binary logistic regression model, with peri-implant condition as the outcome variable, was fit for the data at the implant level, with the patient as the cluster variable; included variables are listed in Table 1.

Because of the small sample size, especially the low within-cluster size of 2.6 implants per patient, in combination with the lack of variability of most level-1 predictors within patients (e.g., multiple implants of the same type inserted at the same time for a specific patient), no random effects or cross level interactions were calculated. As the intra-level interaction effects were non-significant (*p* > 0.1), they were omitted, and only the main effects were interpreted. The marginal effect was estimated without separately analyzing the contextual- and within-patient effects.

Before interpreting the final model, a random intercept-only “null”-model was specified to determine the variance partition coefficient (VPC) that shows the percentage of variance observed in the “peri-implant condition” that can be attributed to patient-specific effects as compared to implant-specific effects. In all models, the Satterthwaite approximation for unbalanced sample size was used.

For the comparison of BOP frequencies at different implant sites, a three-level logistic regression model was utilized, with probing position as the fixed factor. The significance level for the tests was set at 0.05. the results were noted as odds ratios (OR) with 95% confidence intervals (95%CI). The *p*-values were adjusted for multiple comparisons using the sequential Bonferroni correction. The IBM SPSS 27.0 software (IBM, Armonk, NY, USA) was used for the analysis.

## 3. Results

After the primary screening of patient records and radiographs, 154 patients, with a total of 362 implants, were selected on the basis of their radiographs (Figure 1). After further screening and informed written consent had been obtained, 83 patients, with a total of 213 implants, agreed to participate in the study, agreeing to submit to clinical examination and evaluation of the existing current radiograph (Figure 1, Table 2).

All patients were in good general health, the age of the patients at the time point of insertion was between 32 and 76 years (mean 55.8 years (SD 9.27)), and 43 of the patients were female (Table 2). All patients had received periodontal therapy before implant therapy, and no implant was inserted if the patient exhibited a PPD ≥ 5 mm. There were eleven smokers, and all other patients were non-smokers or had not smoked since implantation. All of the smokers were found in the group of patients with residual pockets.

At the examination time point, 44 patients were diagnosed with generalized stage III periodontitis, and 39 patients were diagnosed with stage IV generalized periodontitis. The mean FMPS was 25.3% (SD 13.43), and the mean FMBS was 16.2% (SD 9.10) at the examination time point. Each patient was eligible to participate in a regular maintenance program, but 12 patients (14.5%) did not take this opportunity to maintain their peri-implant and periodontal health. At the examination time point, 12 patients were diagnosed with successfully treated stable periodontitis, meaning that there were no sites of ≥4 mm exhibiting BOP [30]. A total of 9 patients were diagnosed as successfully treated, but with some gingival inflammation, and 62 patients possessed residual pockets with PPD ≥ 5 mm and BOP [30]. The mean number of residual teeth was 18.7 (SD 4.88) teeth/patient (range 7–27; median 19) (Table 2).

No implant was lost in the examined patients at any time point, either in the early healing phase or in the long-term observation follow-up time.

The mean number of implants was 2.6 implants/patient (range 1–10 implants; median 2), and the mean follow-up time was 8.7 years (SD 2.54). The mean max. CAL/implant (study site) was 4.5 mm (SD 1.22; range 1–10 mm; median 4 mm), and at this site, the mean probing depth (PD) was 3.7 mm (SD 1.30; range 1–9 mm; median 3.5 mm). All supraconstructions were cemented and were either single crowns or bridges.

At the examination time point, there were 20 patients (24.1%) exhibiting healthy peri-implant conditions around all their implants (32 implants; implants/patient: mean 1.6; range 1–3 implants; Table 2 and Table 3).

The distribution and combination of implants among patients in relation to peri-implant diagnosis is shown in Table 3. Obviously, patients with the combination of all three diagnoses showed the highest mean of implants/patient. On a patient level, eight patients exhibited an implant with peri-implantitis (Table 2). Of those eight patients, seven showed unstable periodontal conditions [30], seven had implants with an IDRA score of 3, five patients exhibited periodontitis in stage IV, and the mean number of residual teeth of those eight patients was 16.3 (range 10–21); one patient was a smoker, and two did not show up for the SPT. On the implant level, healthy peri-implant conditions were found around 79 of the implants (37.1%), distributed in 53 patients. Peri-implant mucositis was found around 125 implants in 62 patients, and peri-implantitis was noted around 9 implants in 8 patients (Table 4).

The measurement of DIM [23] yielded 102 implants with the implant shoulder/abutment visible (DIM+), 104 implants with the mucosal margin situated at the implant shoulder/abutment (DIM = 0), and seven implants with the mucosal margin situated coronal of the implant shoulder/abutment (DIM-).

The “low risk” IDRA score was not found for any implant in the present study, a “moderate risk” IDRA score was found for 68 implants in 29 patients, and the other 145 implants yielded a “high risk” score in 54 patients. The calculation of the IDRA [19] was patient-specific in our study, with zero variation within patients. The latter was mainly because of their history and stage of periodontitis, their FMBS, residual pockets, and periodontal bone loss in relation to the patient’s age.

The comparison of teeth-related risk parameters for peri-implantitis “% of BOP at teeth” and “% of residual pockets at teeth ≥5 mm” with implant related BOP exhibited significant associations. The correlation between “% of BOP at teeth” and “% of BOP at implants” was positive, with a small effect size (Kendall-Tau-b = 0.197; *p* = 0.011; n = 83) and also between “% of residual pockets at teeth ≥5 mm” and “% of BOP at implants” (Kendall-Tau-b =0.163; *p* = 0.04; n = 83).

Because of the small class sizes for some factors, like smoking status (only 11 were smokers) or SPT compliance (12 patients were non-compliant), the bivariate logistic regression modeling “peri-implant condition” was potentially underpowered to detect relevant differences in those traits or interactions with other predictors.

In the present study, at the patient level, no significant effect of smoking status on the risk for observing peri-implant disease was found, nor did the frequency of SPT or FMPS exhibit any statistically significant effect on the health of peri-implant tissues. However, having more than two implants per patient was significantly related to the risk of patients being diagnosed with peri-implant inflammation (*p* = 0.007; OR 22.9 95%CI 2.3 to 226.7). This was to be expected, as having one implant exhibiting inflammation defined the outcome at the patient level, and a higher number of implants will thus, by chance alone, increase the risk for a higher patient inflammation status.

Analysis at the implant level revealed a variance partition coefficient (VPC) of 0.126, meaning that approximately 13% of the observed variation in the outcome at the implant level could be attributed to patient-specific factors. The overall predicted probability for an implant to exhibit peri-implant inflammation in a typical patient was 62% [95%CI: 53% to 69%] in this study. The probability to develop BOP at more than one sampling point per implant was 36% [95%CI: 27% to 45%] for a typical implant in this study.

As for the analysis at the patient level, having more than two implants per patient was associated with a 2.3 [95%CI 1.04 to 5.1] times increased risk for observing peri-implant inflammation (*p* = 0.041). This time, however, the effect was measured at the implant level and cannot be attributed to aggregation effects. No other predictor at the patient and within-patient level exhibited a significant relationship with the peri-implant condition. Even though the effect size was notably large, the chances for observing peri-implant inflammation were only non-significantly higher for implants in patients with a plaque index > 20% (OR 1.88; 95%CI 0.9 to 3.9; *p* = 0.092) compared to those with PI < 20%, indicating that the statistical power was too small to detect important effects in this real-world setting, despite the considerable sample size.

**Figure 2:**
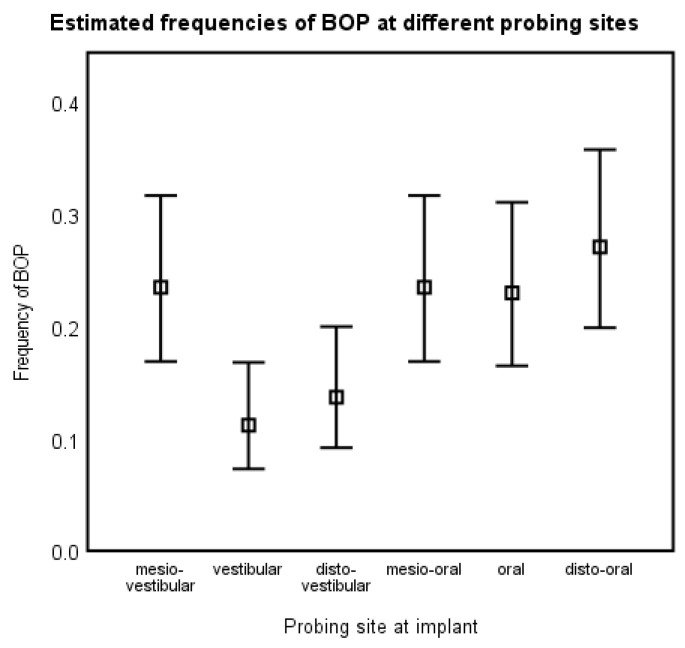
Estimated frequencies of BOP at different probing sites at the implants.

BOP was measured at six sites around each implant (mesio-vestibular, vestibular, disto-vestibular, mesio-oral, oral, and disto-oral). Interestingly, the frequency of BOP was systematically different, depending on the position of the site. At the disto-vestibular (mean frequency: 11.2% [7.3 to 16.8]) and mesio-vestibular (13.7% [9.2 to 20]) measurements, a noticeably lower number of positive sites than those obtained from all four remaining measurement points (mean frequency 24.3 [20.9 to 28.4] was found (multilevel binary logistic regression; comparisons *p* < 0.05; Figure 2). To put it more clearly: the chances for BOP were around two times higher at other sites when compared to the disto-vestibular (OR 2.4 [1.4 to 4.0]; *p* = 0.001) or central vestibular sites (OR 1.9 [1.2 to 3.1]; *p* = 0.013). There was no interaction between the probing site and the implant position (*p* > 0.5).

## 4. Discussion

The results of the present long-term analysis show a high prevalence of peri-implant disease in well maintained patients that had previously been treated for advanced periodontitis. These findings illustrate the challenges involved in the long-term maintenance of oral health in stage III/IV patients restored with dental implants, even for practices specialized in periodontics and implant dentistry. The findings are important, given the high prevalence of severe periodontitis and the increasingly high number of implants being inserted to restore aesthetics and function in these patients. Moreover, there is a scarcity of data available regarding the performance of these treatments in the “real-life situation” of private practice.

The present study exhibits both strengths and limitations. It is certainly a strength that the patient sample is rather homogenous. All of the patients had presented with a history of successfully treated periodontitis before the implants were inserted. All implants were placed by one specialist in the same dental office, under the same conditions, using the same kind of implant. The majority of patients participated in an individual SPC program and were non-smokers or past-smokers. The length of the follow-up (5–17 years) can be seen as another advantage.

On the other hand, these facts limit the generalizability of the results, as not all patients affected by the sequelae of stage III/IV periodontitis can benefit from such an ideal setting. Moreover, the retrospective approach has some inherent limitations: only a selected sample of patients from the practice could be included. It has to be assumed that patients that were invited but did not participate in the follow-up examination were less interested and less compliant and would have probably presented with worse conditions than did those patients included in the present analysis. Thus, it is likely that the findings in the present “selected patient sample” were significantly better than the outcomes of the whole sample of patients treated in the practice would have been.

In the present study, the likelihood that an implant would experience peri-implant disease was more than two times (OR 2.3 [95%CI 1.04 to 5.1]) higher when the total number of implants in one patient was higher than two. A correlation between the number of implants/patient and peri-implant disease may be based on the fact that people who have lost their teeth due to periodontitis have a higher susceptibility to peri-implant inflammation [6,12,19]. That is, the higher the tooth loss, the higher the number of implants. In this context, however, it is surprising that the number of lost teeth/patient was not a significant predictor of peri-implant inflammation.

Peri-implant mucositis is a reversible condition, but it is seen as a risk for developing peri-implantitis [16,27,31]. In the present study, 79 implants (37.1%) were diagnosed as peri-implant healthy, while only 20 (24.1%) presented with peri-implant health at all implants (Table 2). In the very recently published EFP S3 level guideline “Prevention and Treatment of Peri-Implant Diseases”, on a patient basis, the prevalence of peri-implant mucositis was 43–47%, and for peri-implantitis it was 20–22% [16]. In the present study, at the patient level, a higher prevalence for peri-implant mucositis (66.3%) and a lower prevalence for peri-implantitis (9.6%) was diagnosed. This may be a result of the low number of smokers and low numbers of non-compliant patients included in the present study. All patients visited the same surgeon/periodontist over many years; thus, BOP on an implant/tooth would, of course, be treated according to the state of the art. Moreover, the specialized practice had a clear opinion about smoking; the population from this practice may thus be influenced by or selected according to this approach. Only about 13% of patients in the present study were smokers, and all other included patients had either never smoked or had quit smoking at the time of implantation and had not started smoking again.

The major part of the included implants in the present study had a diagnosis of peri-implant mucositis (66.3%), 9.6% showed signs of peri-implantitis, with inflammation and bone loss extending the rate of remodeling. This emphasizes the importance of SPT on a regular basis, as also stated in the Evidence-Based Guideline for Peri-Implant Therapy from the XVIII European Workshop on Periodontology [16,32]. Most patients in the present study were involved in regular maintenance, 71 of the included 83 patients took the opportunity to visit the dental office on a regular basis. The number of patients not keeping their appointments for SPT was relatively low, and no conclusion as to the influence of regular maintenance on the results was possible.

Interestingly, comparing the six sites around an implant where measurements were carried out, the sites were unequal in their frequency of bleeding on probing (Figure 2). There was a dependency on the location of the site of measurement. Disto-vestibular and vestibular locations showed the significantly lowest tendency to bleed. With regard to the central vestibular site, this finding is perhaps not so spectacular, as this is perhaps the easiest site for patients to access for self-cleaning. It is interesting, however, that the disto-vestibular site also showed less inflammation, because the patient’s view of this site is blocked by the implant itself. One explanation may be that the patient can better clean this area using an interdental device, since the movement and pressure of the interdental device is naturally pressed forward (against the implant). At the mesial site of the implant, it might perhaps be (visual) easier to place the interdental device, but it will have to be pressed backwards against the implant in the distal direction.

The influence of a mild to severe inflammation on the probe penetration in sulci around the implants was investigated in Macaca monkeys, but not the dependence of the site [33]. In that study, an association with deeper probe penetration and inflammation around the implants in comparison to teeth with inflammation in the surrounding periodontal tissues was shown. This illustrates the extreme importance of patients’ home care in regards to their implants. It also shows the importance of monitoring BOP, as a sign of inflammation—not only to diagnose existing conditions, but also enable timely intervene in order to maintain periodontal and peri-implant health. In addition, these findings emphasize the importance of recording PPD and BOP at all six sites around the teeth and the implants as standard procedure in SPT [12,34]. Another finding was the percentage of BOP around teeth. This correlated significantly with a higher bleeding on probing around implants, but only with a small effect. 

All patients included in the present study had a diagnosis of periodontitis in Stage III or IV. One major risk factor for the survival of an implant is the history of periodontitis [6,12,19]. In daily practice, implants with peri-implant inflammatory changes might be associated with suboptimal conditions during implantation, like uncontrolled periodontitis [12]. Implants were previously recognized as a substitute for periodontally compromised teeth, regardless of the periodontal condition of the patient; this assumption is based on a 1995 study reporting a high survival rate of implants in patients with refractory periodontitis [35]. In that study, two surgeons had inserted 309 implants, and the time in function of the implants was 2–8 years. But, of these implants, 250 (80.9%) had only been in function for a maximum of 5 years. In the present long-term study, all examined implants had been in function for more than 5 years in patients with a history of periodontitis.

In the present study, no implant was lost, even though all patients had a history of periodontitis at the time of implant insertion. However, no implant was inserted if PPD > 4 mm. A thorough periodontal treatment was carried out before implantation. Although the rate of peri-implant inflammation was relatively high over the 5 years after implantation, only 9.6% of the implants in eight patients were diagnosed with peri-implantitis. This is significantly lower than the numbers of 20–22% reported in the very recently published EFP S3 level guideline [16]. Recently, a systematic review addressed implant therapy in partly edentulous patients with periodontitis based on 17 randomized clinical controlled trials [36]. The patients in the included trials were treated with implant-supported fixed partial dentures and had implants in function between five to ten years, similar to the patients in the present study. A higher risk for peri-implantitis and a poorer implant survival rate was associated with a history of periodontitis. A very recently published systematic review from the XVIII European Workshop on Periodontology stressed the importance of regular periodontal/peri-implant care as a primary prevention measure [37].

Patients who have a history of severe periodontitis, poor plaque control, and no regular maintenance care are identified as having a greater risk for developing peri-implantitis [12]. In addition to the history of periodontitis, the daily oral hygiene of the patients was also monitored. The significant influence of poor oral hygiene on peri-implant health was shown in several studies [38,39]. In the present study, however, FMPS did not have a statistically significant influence on the diagnosis of the peri-implant tissues, probably because FMPS was relatively low (mean 25.3%). The amount of FMPS is known to influence peri-implant conditions [12], and likely the number of patients in this study with a very low FMPS was too small to show its influence on peri-implant disease. Other confounding factors for the development of periodontal and peri-implant diseases are smoking and the lack of participation in a supportive program (SPT) [6]. In a recently published narrative review, the use of postbiotics, ozone, air-polishing devices, etc. adjunctive to non-surgical therapy was discussed [15]. The authors concluded that some benefits of adjunctive methods may be suspected. However, due to low numbers of studies investigating this, no firm conclusions can be drawn [15].

To achieve long-term peri-implant health, especially in periodontally compromised patients, it is important for any practice specializing in periodontology and implantology to emphasize smoking cessation and the willingness to maintain good plaque control, in combination with regular visits for risk assessment and supportive care (SPT) [6]. However, in the present study, the number of smokers and non-compliant patients was too small to establish statistically significant relationships with peri-implant health or disease. Apparently, the practice had either selected mainly compliant patients in the first place, or had successfully convinced its patients to adhere to plaque control measures and to quit smoking (and not resume this activity) at the time of implantation.

The interesting point of the present study is that this is a study using data from a specialized dental practice, including its daily challenges, and not a study from a university. This was already a point of discussion in 2008, when the need for further research was proposed at the VI European Workshop on Periodontology [18].

## 5. Conclusions

The results of the present long-term analysis show a high prevalence of peri-implant disease in patients that had been treated for advanced periodontitis, despite the fact that the vast majority of them had shown good compliance with regular SPC. These findings illustrate the enormous challenges involved in the long-term maintenance of oral health in stage III/IV patients restored with dental implants, even for practices specializing in periodontics and implant dentistry.

## Figures and Tables

**Table 1:** Variables included in the final multilevel model predicting peri-implant condition at the implant level.

Level	Variables	Values	
Patient	Sex	male	female
	Smoke status	non-smoker/former smoker	smoker
	SPT compliance	non/infrequently	frequently
	FMPS	≤20%	>20%
	# lost teeth	count	
	# implants	≤2 implants	>2 implants
Implant	Implant type	Straumann® Standard	Straumann® Standard Plus
	Implant surface	SLA	SLAactive
	Years in function	years	
	Patient age at insertion	years	
	Implant location	mandible	maxilla
	*Outcome variable*: peri-implant condition	healthy	inflammation

FMPS: Full Mouth Plaque Score [22].

**Table 2:** Demographic data of patients at examination time point.

Variable	Outcome
Patients	83
age at insertion (years; mean (SD); range)	55.8 (9.27); 32–76
men/women (n)	40/43
mean residual teeth per patient (mean (SD); range)	18.7 (4.88); 7–27
implants (n)	213
mean implants per patient (mean (SD); range)	2.6 (1.87); 1–10
Smoking habits (n/% (pack years))	
never smoked or former smoker (>5 years)	72/86.7%/(-)
smoker	11/13.3% (32.2)
Periodontal condition (n patients/%) [30]	
Treated periodontitis patients with stable conditions	12/14.5%
Treated periodontitis patients with some inflammation	9/10.8%
Treated periodontitis patients with unstable conditions	62/74.7%
Implant-based diagnosis (n patients/%) [26,27,28]	
patients with all implants showing peri-implant healthy conditions	20/24.1%
patients with at least one implant with peri- implant mucositis	55/66.3%
patients with at least one implant with peri- implantitis	8/9.6%
FMPS (%; mean (SD)) [22]	25.3 (13.43)
FMBS (%; mean (SD))	16.2 (9.10)
Maintenance (SPT; n/%)	
no or sporadic maintenance	12/14.5%
regular maintenance	71/85.5%

FMPS: Full Mouth Plaque Score; FMBS: Full Mouth Bleeding Score [22].

**Table 3:** The distribution and combination of implants among patients in relation to peri-implant diagnosis.

Peri-Implant Diagnosis	Patients	Implants	Mean Implants/Patient
Healthy Peri-implant mucositis only	20	32	1.6
	27	48	1.8
Peri-implantitis only	1	1	1
Combination health and PI-M	28	97	3.5
Combination health and P-I	1	2	2
Combination PI-M and P-I	2	7	3.5
Combination of all 3 diagnoses	4	26	6.5
Total	83	213	

PI-M: peri-implant mucositis; P-I: peri-implantitis.

**Table 4:** Implant characteristics at examination time point.

Variable	Outcome
Implant in function (years; mean (SD), range)	8.67 (2.57), 5–16
5–10 years (n/%)	136/63.8%
≥10 years (n/%)	77/36.2%
Position (n/%)	12/14.5%
maxilla	9/10.8%
mandible	62/74.7%
Distribution of implants in regards to the surface in jaws (n/%)	
maxilla/SLA^®^ surface	76/35.7%
maxilla/SLActive^®^ surface	75/35.2%
mandible/SLA^®^ surface	35/16.4%
mandible/SLActive^®^ surface	27/12.7%
Implant diagnosis (n/%) [12]	
implants with healthy peri-implant conditions	79/37.1%
implants with peri-implant mucositis	125/58.7%
implants with peri-implantitis	9/4.2%
Implant Disease Risk Assessment (IDRA) [19] (n implants/%)	
IDRA score “low risk”	0/0%
IDRA score “moderate risk”	68/31.9%
IDRA score “high risk”	145/68.1%

## Data Availability

The data that support the findings of this study are available from the corresponding author upon reasonable request.
